# Supplementary material for: Milk Intake in Early Life and Later Cancer Risk: A Meta-Analysis
Source: Nutrients. 2022 Mar 15;14(6):1233. doi: 10.3390/nu14061233 (PMC8948718; doi:10.3390/nu14061233)

List of supplementary materials:

1. Supplementary Table S1: MOOSE Checklist
2. Supplementary Table S2: Database Search Strategy
3. Supplementary Table S3: Characteristics of Studies Included
4. Supplementary Table S4: Meta-analyses of dairy intake in early life with any breast cancer risk
5. Supplementary Table S5: Meta-analyses of dairy intake in early life with any prostate cancer risk
6. Supplementary Table S6: Meta-analysis of dairy intake in early life with any colorectal cancer risk
7. Supplementary Figure S1: Funnel plot for the meta-analysis of milk intake during early life with any breast cancer risk
8. Supplementary Figure S2: Contour-enhanced funnel plot for the meta-analysis of milk intake during early life with any breast cancer risk, applying the trim-and-fill method

Supplementary Table S1. MOOSE Checklist

Criteria		Brief description of how the criteria were handled in the meta-analysis
**Reporting of background should include**		
√	Problem definition	Reported on page No. 1-2
√	Hypothesis statement	Reported on page No. 1-2
√	Description of study outcomes	Breast, prostate, colorectal cancer risk later in life
√	Type of exposure or intervention used	Milk intake in childhood and adolescence
√	Type of study designs used	We included observational studies.
√	Study population	We placed no restriction.
**Reporting of search strategy should include**		
√	Qualifications of searchers	The credentials of the investigators were indicated in the author list.
√	Search strategy, including time period included in the synthesis and keywords	Search was done to include studies published through December 2021.<br><br>Detailed search strategy was provided in Supplementary Table S1.
√	Databases and registries searched	PubMed and Embase
√	Search software used, name and version, including special features	We did not employ a search software.<br><br>EndNote was used to merge retrieved articles and eliminate duplications.
√	Use of hand searching	The reference lists of all the articles included in this analysis were also reviewed for additional studies.
√	List of citations located and those excluded, including justifications	Details of the literature search process are outlined in Figure1.
√	Method of addressing articles published in languages other than English	We restricted the language to English.

√	Method of handling abstracts and unpublished studies	We excluded abstracts and unpublished results.
√	Description of any contact with authors	No author contact was made for this manuscript.
**Reporting of methods should include**		
√	Description of relevance or appropriateness of studies assembled for assessing the hypothesis to be tested	Reported on page No. 2-4
√	Rationale for the selection and coding of data	Reported on page No. 2-3
√	Assessment of confounding	We extracted the most appropriate adjusted RRs.
√	Assessment of study quality, including blinding of quality assessors; stratification or regression on possible predictors of study results	Subgroup analyses by menopausal status, cancer stage, fat content of milk, life stage of milk, or study design were conducted.
√	Assessment of heterogeneity	Heterogeneity was quantified by I^2.
√	Description of statistical methods in sufficient detail to be replicated	Reported on page No. 3-4
√	Provision of appropriate tables and graphics	We included 12 figures (flow chart, forest plots). Additional table and figures were also provided in supplementary materials.
**Reporting of results should include**		
√	Graph summarizing individual study estimates and overall estimate	Figure 2-4
√	Table giving descriptive information for each study included	Supplementary table S2
√	Results of sensitivity testing	Supplementary table S4-6
√	Indication of statistical uncertainty of findings	95% confidence intervals were presented for all summary estimates.
**Reporting of discussion should include**		

√	Quantitative assessment of bias	Publication bias was tested via Egger's test.
√	Justification for exclusion	Reported on page No. 2-3<br><br>Non-human studies or articles that do not provide required data for meta-analysis were excluded.
√	Assessment of quality of included studies	Subgroup analyses were conducted by menopausal status, cancer stage, fat content of milk, life stage of milk, or study design, but the results were inconsistent.
**Reporting of conclusions should include**		
√	Consideration of alternative explanations for observed results	Reported on page No. 10-12
√	Generalization of the conclusions	Reported on page No. 12
√	Guidelines for future research	Reported on page No. 12
√	Disclosure of funding source	Reported on page No. 12

Supplementary Table S2. Database Search Strategy

**PubMed**	(Dairy Products[Mesh] OR Dairy[tw] OR milk[tw] OR yogurt[tw] OR cheese[tw]) AND (child[Mesh] OR adolescent[tw] OR adolescence[tw] OR early[tw] OR childhood[tw] OR teens[tw] OR preschool[tw]) AND ("Neoplasms"[Mesh] OR neoplasm[tw] OR neoplasms[tw] OR neoplasia[tw] OR carcinoma[tw] OR carcinomas[tw] OR cancer[tw] OR cancers[tw] OR tumor[tw] OR tumors[tw] OR tumour[tw] OR tumours[tw]) NOT (Case Reports[ptyp] OR Comment[ptyp] OR Letter[ptyp] OR Editorial[ptyp]) NOT (animals[Mesh] NOT humans[Mesh])
**Embase**	('Dairy Product'/exp OR Dairy:ti,ab OR milk:ti,ab OR yogurt:ti,ab OR cheese:ti,ab) AND (adolescent:ti,ab OR adolescence:ti,ab OR early:ti,ab OR childhood:ti,ab OR teens:ti,ab) AND ('malignant neoplasm'/exp OR neoplasm:ti,ab OR neoplasms:ti,ab OR neoplasia:ti,ab OR carcinoma:ti,ab OR carcinomas:ti,ab OR cancer:ti,ab OR cancers:ti,ab OR tumor:ti,ab OR tumors:ti,ab OR tumour:ti,ab OR tumours:ti,ab) NOT ('case report'/de OR 'editorial'/de OR 'letter'/de OR 'review'/de) NOT ([animals]/lim NOT [humans]/lim)

Supplementary Table S3. Characteristics of Studies Included

First author, Year, Country,	Cohort name, study design	Sex, Age at baseline	Age of milk intake	RR for highest vs. lowest milk intake (95%CI)
**Any Breast Cancer**				
Haraldsdottir, 2018, Iceland	Age Gene Environment Susceptibility(AGES), Prospective study	F, 76.5yrs	14-19	0.70 (0.40, 1.10)
Linos, 2010, USA	Nurses' Health Study II, Prospective study	F, 34-53yrs	High school	0.98 (0.71, 1.34)
Knight, 2007, Canada	NA, Retrospective study	F, 20-69yrs	10-19	0.62 (0.45, 0.86)
Pols, 2007, UK	Boyd Orr cohort, Prospective study	F, 8yrs	4-11	0.83 (0.41, 1.69)
Frazier, 2003, USA	Nurses' Health Study I, Retrospective study	F, 40-65yrs	High school	1.01 (0.95, 1.07)
Hjartaker, 2001, Norway	Norwegian Women and Cancer Study (NOWAC), Prospective study	F, 40.7yrs	Child	0.64 (0.22, 1.87)
Shu, 2001, China	The Shanghai Breast Cancer Study, Retrospective study	F, 25-64yrs	13-15	0.76 (0.59, 0.98)

First author, Year, Country,	Cohort name, study design	Sex, Age at baseline	Age of milk intake	RR for highest vs. lowest milk intake (95%CI)
**Any Prostate Cancer**				
Lan, 2020, USA	NIH-AARP Diet and Health Study, Prospective study	M, 50-71yrs	12-13	1.06 (0.99, 1.14)
Torfadottir, 2011, Iceland	Age Gene Environment Susceptibility(AGES), Prospective study	M, 32-60yrs	14-19	1.38 (0.85, 2.25)
Pols, 2007, UK	Boyd Orr cohort, Prospective study	M, 8yrs	4-11	0.41 (0.16, 1.05)
Andersson, 1995, Sweden	NA, Retrospective study	M, 64-76	Adolescence	0.80 (0.50, 1.40)

First author, Year, Country,	Cohort name, study design	Sex (% men), Age at baseline	Age of milk intake	RR for highest vs. lowest milk intake (95%CI)
**Any Colorectal Cancer**				
Cox, 2011, New Zealand	NA, Retrospective study	M/F (51.0) 30-69yrs	5-18	0.39 (0.20, 0.77)
Ruder, 2011, USA	NIH-AARP Diet and Health Study. Prospective study	M/F (58.5) 50-71yrs	12-13	0.84 (0.71, 0.99)

First author, Year, Country,	Cohort name, study design	Sex (% men), Age at baseline	Age of milk intake	RR for highest vs. lowest milk intake (95%CI)
Pols, 2007, UK	Boyd Orr cohort, Retrospective study	M/F (49.4) 8yrs	4-11	2.45 (1.11, 5.41)

Abbreviations: F, Female; M, Male; yrs, years.

Supplementary Table S4. Meta-analyses of dairy intake in early life with any breast cancer risk

	No. of studies	RR (95% CI)	I^2 (%)
**Overall**	8	0.90 (0.78, 1.03)	56
**By menopausal status**			
Premenopausal women	4	0.99 (0.85, 1.16)	0
Postmenopausal women	3	1.00 (0.82, 1.23)	25
**By cancer stage**			
Invasive breast cancer	2	0.83 (0.48, 1.43)	88
Any breast cancer	6	0.93 (0.83, 1.06)	30
**By fat content of dairy**			
Low-fat dairy	2	1.02 (0.90, 1.15)	0
Regular dairy	8	0.87 (0.76, 1.00)	55
**By life stage of dairy**			
10 ≤ Age(years) ≤ 19	6	0.90 (0.77, 1.04)	67
Age(years) <10	2	0.81 (0.46, 1.44)	0
**By study design**			
Retrospective study	4	0.87 (0.71, 1.05)	76
Prospective study	4	0.97 (0.79, 1.20)	7

Supplementary Table S5. Meta-analyses of dairy intake in early life with any prostate cancer risk

	No. of studies	RR (95% CI)	I² (%)
**Overall**	4	1.05 (0.86, 1.29)	23
**By cancer stage**			
Advanced prostate cancer	2	1.12 (0.57, 2.20)	41
Any prostate cancer	4	1.05 (0.86, 1.29)	23
**By life stage of dairy**			
12 ≤ Age(years) ≤ 19	2	1.08 (1.02, 1.15)	0
Age(years) <12	1	0.55 (0.21, 1.43)	NA
**By study design**			
Retrospective study	1	0.80 (0.44, 1.44)	NA
Prospective study	3	1.09 (0.84, 1.40)	31

Supplementary Table S6. Meta-analysis of dairy intake in early life with any colorectal cancer risk

	No. of studies	RR (95% CI)	I^2 (%)
**Overall**	3	1.07 (0.59, 1.94)	77

Supplementary Figure S1. Funnel plot for the meta-analysis of milk intake during early life with any breast cancer risk

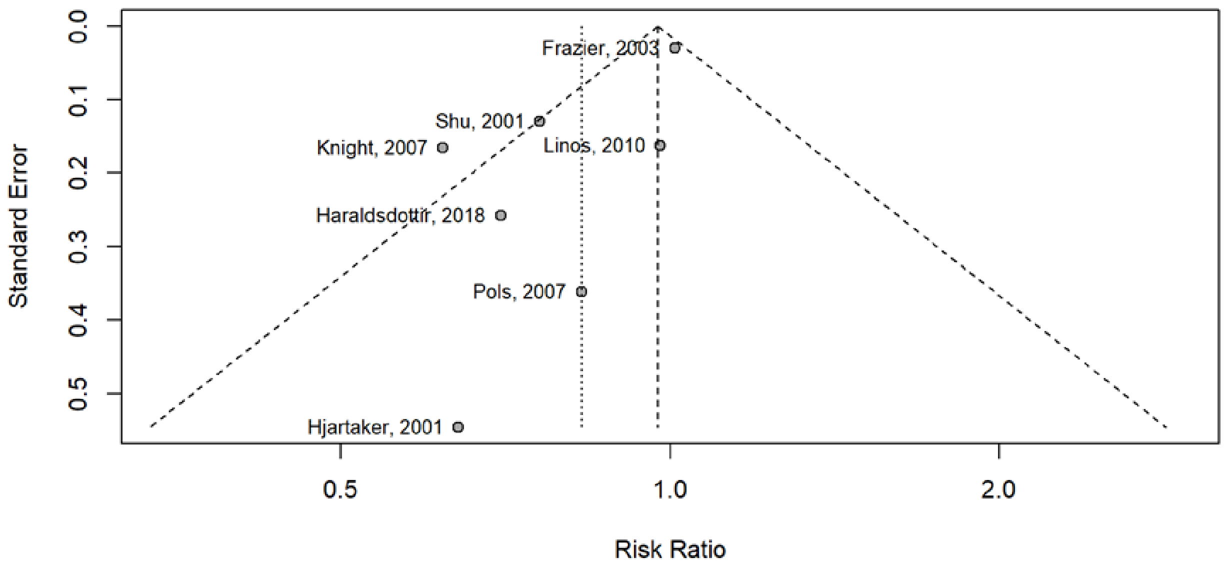

Supplementary Figure S2. Contour-enhanced funnel plot for the meta-analysis of milk intake during early life with any breast cancer risk, applying the trim-and-fill method

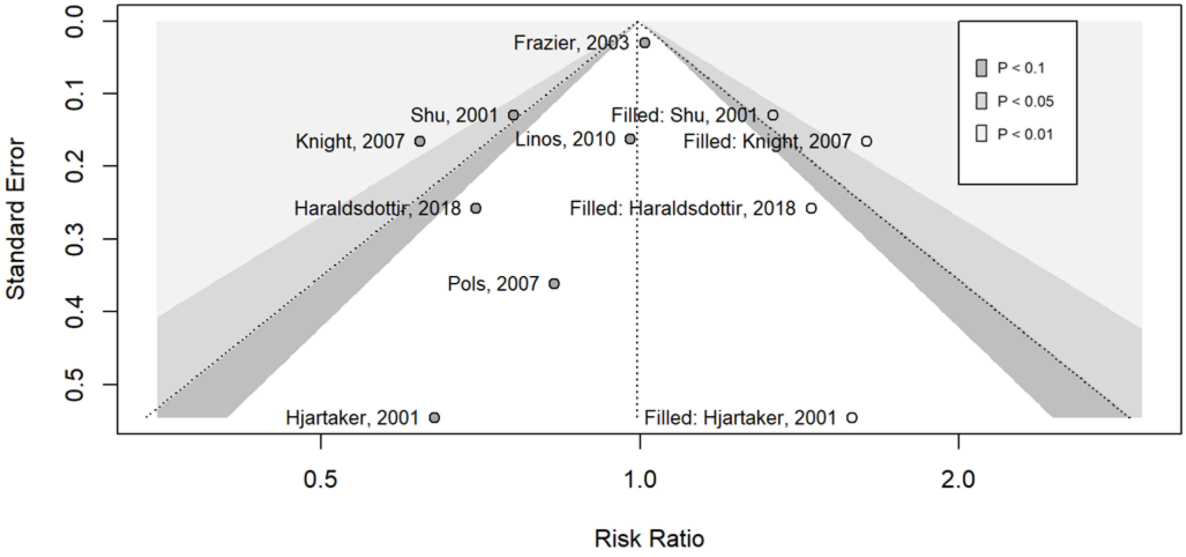

Supplement: Supplementary file 1 [file nutrients-14-01233-s001.zip › nutrients-1606882-supplementary.pdf]
